# Embodied Cognition in Meditation, Yoga, and Ethics—An Experimental Single-Case Study on the Differential Effects of Four Mind–Body Treatments

**DOI:** 10.3390/ijerph191811734

**Published:** 2022-09-17

**Authors:** Karin Matko, Peter Sedlmeier, Holger C. Bringmann

**Affiliations:** 1 Institute of Psychology, Chemnitz University of Technology, 09120 Chemnitz, Germany; 2 Institute of Social Medicine, Epidemiology and Health Economics, Charité—Universitätsmedizin Berlin, Corporate Member of Freie Universität Berlin and Humboldt-Universität zu Berlin, 10117 Berlin, Germany; 3 Department of Psychiatry and Psychotherapy, Krankenhaus Spremberg, 03130 Spremberg, Germany

**Keywords:** embodied cognition, yoga components, mantra meditation, body awareness, emotion regulation, differential effects, single-case research, long-term effects

## Abstract

Yoga is an embodied contemplative practice considered as a path toward long-term well-being, which fosters an integrated processing of bodily and emotional stimuli. However, little is known about how the different components of yoga contribute to these processes. This was the aim of this single-case multiple-baseline study. Herein, we explored how different yoga components affect body awareness, emotion regulation, affectivity, self-compassion, and distress tolerance. Forty-two randomly assigned participants (from initially fifty-seven) completed one of four 8-week treatments: Mantra meditation alone (MA), meditation plus physical yoga (MY), meditation plus ethical education (ME), and meditation plus yoga and ethical education (MYE). Participants had no prior regular yoga or meditation practice. Data were analyzed using visual inspection, effect size estimation, and multilevel modeling. Surprisingly, all four treatments similarly improved body awareness (Tau-*U_MA_* = 0.21 to Tau-*U_MY_* = 0.49), emotion regulation (Tau-*U_MYE_* = −0.43 to Tau-*U_ME_* = −0.52), self-compassion (η^2^ = 0.08), and distress tolerance (η^2^ = 0.13). These effects were maintained until follow-up at 2 and 12 months after the study, even though home practice declined. The MA condition had the least favorable effect on affective experience (Tau-*U_MA_* = −0.14 and 0.07), while the ME condition enhanced valence the most (Tau-*U_ME_* = 0.10) and the MY condition was the most effective in preventing negative affective responses. Although mantra meditation on its own negatively influenced daily affect, it can be assumed as the driving force behind the improvement in the other variables. This points to the central role of meditation in increasing interoception, self-awareness, and embodied processing.

## 1. Introduction

Yoga has been proposed as an embodied intentional practice and path toward eudaimonic well-being and a well-lived life of fulfilled meaning and purpose [1]. Theories on “embodiment” or “embodied cognition” assume a complex reciprocal relationship between bodily states, emotion, cognition, and behavior [2,3,4]. Nevertheless, the body is still overlooked in positive psychology, despite its undeniable interconnection with the overall experience of well-being and flourishing [5]. On the other hand, yoga is one of the most renowned mind–body exercises, which promotes engagement with body sensations and awareness [6]. Along with other mind–body practices, it has been proposed to connect body and mind and facilitate the integration of both bottom-up physiological and top-down cognitive processes [7,8,9]. These practices might enhance practitioners’ experience of unity between the body and the self, or of an “embodied self” [10]. However, traditional yoga encompasses a multiplicity of components [11], and little is known about which differential health benefit is associated with which yoga component.

Gard et al. [8] posited that the ethical component of yoga encourages the top-down initiation, monitoring, and maintenance of behavioral change, whereas postures, breathing regulation, and meditation stimulate top-down attentional control as well as bottom-up interoceptive processing. Schmalzl et al. [9] delineated how the movement-related aspects of yoga positively influence the neurocircuitry involved in mind–body integration and self-regulation. On the other hand, yogic breathing improves autonomic regulation and executive functioning, and meditation increases interoceptive and metacognitive awareness. However, there have been only few comparative or dismantling studies investigating explicitly how each of these components or their combinations work [12]. Specifically, the ethical component of yoga has often been neglected in the past [13]. In addition, there is a scarcity of studies examining the differential effects of different yoga components on processes of embodied cognition.

Moreover, embodied cognition is not a uniform concept and theories differ regarding the processes it entails [14]. Some of the processes labeled as embodied are body awareness [6], emotion and affect [15], emotion regulation [16], compassion [17], and stress [18]. Deficits or problems in emotion regulation, body awareness, or an embodied sense of self are associated with a variety of forms of psychopathology [19,20,21]. Furthermore, this applies to two other embodied processes closely related to emotion regulation. Self-compassion and distress tolerance have been identified as important protective factors of mental health [22,23] and possible mediators of yoga interventions [24,25].

Body awareness denotes the sensory awareness of bodily states, processes, and actions, including both proprioception (perception of self-movement and body position) and interoception (perception of internal bodily signals) [26]. It is closely related to feelings, emotions, and affectivity [27,28]. Research has shown that effective emotion regulation is highly dependent on a person’s ability to accurately detect and evaluate sensory information [29,30,31]. Emotion regulation can involve different regulatory strategies, such as suppression of expressive behavior, attentional control, cognitive reinterpretation (reappraisal), or reversal of stimulus-response associations (extinction) [32]. Recently, Chambers et al. [33] proposed that the practice of mindfulness might train another type of regulatory strategy, namely, non-reactively observing all emotions and refraining from any appraisal processes. This type of emotion regulation is particularly closely related to body awareness. However, it remains unclear how body awareness and emotion regulation develop over time and how they influence daily affective experience when a person engages in contemplative practices, such as yoga. Interestingly, emotion regulation was associated with top-down strategies (e.g., reappraisal) in novice meditators, but with bottom-up strategies (e.g., interoceptive awareness) in long-term meditators [34]. 

There are only few comparative studies examining the differential impact of yoga components on embodied processing. Park et al. [35] contrasted a complex yoga intervention to a cognitive-behavioral stress-management course and found that only yoga improved interoceptive awareness and emotion regulation. Sauer-Zavala et al. [36] juxtaposed the body scan, sitting meditation, and physical yoga and found that all treatments improved self-compassion, but the latter two were more effective in improving emotion regulation. Kok and Singer [37] examined four different types of meditation and discovered that all practices enhanced positivity of affect; however, the body scan led to the greatest state increase in interoceptive awareness. In a cross-sectional survey, yoga practitioners reported higher levels of body awareness compared to meditators; however, both groups did not differ regarding emotion regulation [38]. For well-being, stress, depression, and anxiety, the treatments combining several yoga components often yielded better results than simple treatments [12]. Nevertheless, it remains unclear which component is associated with which specific benefit, or which combination of practices might be the most helpful, particularly regarding embodied processing. Therefore, a detailed investigation of yoga components is imperative for advancing scientific research. Furthermore, this endeavor is valuable for developing personalized interventions taking into account the specific needs of clinical or healthy populations [9,39]. 

In addition to the lack of differentiation and systematic investigation, the methodological quality of yoga studies has often been poor [40,41]. Dismantling or comparative studies with several repeated measurements, such as experience sampling or single-case research designs [42,43], provide a promising approach in this regard. Therefore, in the present study, we used a single-case multiple-baseline design to evaluate four different combinations of yoga components. The components were based on the new mind–body program Meditation-Based Lifestyle Modification (MBLM) [44]. We chose this design as it has strong internal validity and enables researchers to investigate in-depth how change processes develop over time and across different individuals [45]. This makes it highly suitable for explorative research. With this approach, we hope to determine which combination of components might be particularly beneficial for improving body awareness, emotion regulation, affectivity, self-compassion, and distress tolerance in healthy participants. 

From theory and the literature reviewed above, we assume differential effects for different combinations and stronger (incremental) effects for combined interventions. It seems plausible that both physical yoga and meditation, and specifically their combination, enhance our variables of interest. The physical yoga component might even have a slightly greater impact on body awareness than meditation alone. Ethical education, and specifically its combination with other practices, might be particularly beneficial for developing emotion regulatory skills [46]. However, as we know little about how different mind–body interventions differentially and incrementally affect processes of embodied cognition, we chose an exploratory approach. Combining quantitative and qualitative methods, we examined interindividual differences, incremental differences between conditions, and interactions between our dependent variables.

## 2. Method

### 2.1. Procedure

The present study is a secondary analysis of data that was previously reported by Matko et al. [47,48]. It expands the previously reported findings by providing a detailed analysis of affectivity, body awareness, emotion regulation, distress tolerance, and self-compassion. The study employed a single-case multiple-baseline design to investigate the effects of different combinations of yoga practices over the course of four different treatments. Participants went through a baseline phase, where they received no treatment, and an 8-week treatment phase. The baseline phase could be 7, 14, or 21 days and treatments could be mantra meditation alone (MA), mantra meditation plus physical Hatha yoga (MY), mantra meditation plus ethical education (ME), and mantra meditation plus physical yoga and ethical education (MYE). The baseline length was determined by balancing the need for repeated measurements and the anticipated response burden of our participants. We randomized participants across these conditions and baseline lengths using a random number generator. Participants completed short daily online questionnaires throughout the entire study and longer online questionnaires at pre-test, post-test, and follow-up (8 weeks and 12 months). The study methods and treatments are described in more detail by Matko et al. [47]. 

### 2.2. Participants

Fifty-seven participants were recruited from the Dresden general community through public and web-based advertising. Forty-two participants, 83% women, *M* (*SD*) age = 26.62 (8.37), completed the treatment and were included in the analysis. Individuals were excluded from participation if they had pre-existing psychiatric conditions or acute psychological issues, had a regular yoga or meditation practice during the last 6 months, were younger than 18 years, or did not have daily access to web-enabled devices. Participation was voluntarily and all participants provided written consent to participate in the study. The nature of the study was fully disclosed to participants before randomization and data collection, but they were not allowed to choose their treatment. Participants received no financial or other compensation for participating in the study. The institutional review board of the Chemnitz University of Technology approved the experimental protocol. We analyzed the data of all 42 participants on the daily measurements. Of all participants, 41 completed the post-test, 40 the 2-month follow-up, and 33 the 12-month follow-up. For these data, we performed the analyses only on complete datasets.

### 2.3. Treatment

HCB, an accredited psychiatrist and psychotherapist, and KM, a psychologist and certified yoga instructor with 8 years of teaching experience, jointly led all treatments. The four treatments differed in the length of their weekly meetings due to the difference in the included components (total length MA: 60 min, MY: 105 min, ME: 135 min, MYE: 180 min). However, in each condition, the weekly sessions started and ended with the group sharing their experiences and included a collective 25-min silent mantra meditation practice. In the treatments that included Hatha yoga, participants practiced simple breathing techniques, the sun salutation, relaxation techniques, and a set of simple yoga postures. During ethical education, participants learned about and discussed the 10 yogic Yamas and Niyamas, following the protocol of the MBLM mind–body program [44]. In addition, and according to their treatment, participants should daily practice 20 min of mantra meditation, 20 min of yoga exercises, and/or engage in mindful living activities related to the ethical topic of the week.

### 2.4. Measures

All measures were obtained online using SoSci Survey [49]. The measures that were collected daily or every few days had to be suitable for repeated measurement and, thus, be short and sensitive to changes. For this reason, we had to adapt the established questionnaires for body awareness and emotion regulation. 

We measured body awareness two times per week, and, for this purpose, developed a new questionnaire by extracting the 11 most suitable items from four questionnaires (FFMQ; [50], MAIA; [51], PBCS; [52], BAQ; [53]). This questionnaire was thoroughly tested in two pilot studies. The final set of items can be found in Supplementary Material—Table S1. We used a shortened version of the Difficulties in Emotion Regulation Scale (DERS; [54]) to weekly measure emotion regulation. This scale is a well-validated and widely used self-report measure with excellent psychometric properties (internal consistency α = 0.93). Higher scores indicate higher levels of emotion *dys*regulation. As the DERS is a very long scale for regular measurements, we decided to shorten it by extracting the two items with the highest factor loadings per subscale, resulting in 12 items altogether (see Supplementary Material—Table S2). The daily affective experience of participants was assessed with the economic single-item measure Affective Grid [55], which was specifically developed for repeated observations. The Affective Grid assesses affect along the two dimensions, pleasure-displeasure and arousal-sleepiness, and has shown adequate reliability and validity [56]. 

We measured distress tolerance with the Distress Tolerance Scale (DTS; [57]). It is a 15-item self-report questionnaire examining the degree to which individuals experience negative emotions as intolerable. The DTS has shown good internal consistency (α = 0.89). We assessed self-compassion with the German version of the Self-Compassion Scale (SCS-D; [58]). It evaluates a person’s ability to be kind and forgiving to themselves in difficult circumstances. The SCS has been shown to be valid and reliable. To reduce response burden, we decided to shorten the original 26-item scale by again extracting the two items with the highest factor loadings per subscale, reducing it to 12 items (see Supplementary Material—Table S3). Distress tolerance was measured at pre-test, post-test, and 2-month follow-up. Self-compassion was assessed at pre- and post-test. Body awareness and emotion regulation were measured daily during the main study period and once at 2- and 12-month follow-up. For consistency reasons, all questionnaires (except for the Affective Grid) were rated on a 5-point Likert scale. 

At the beginning of the treatment phase, the daily questionnaire included questions that measure participants’ home practice. They were asked to indicate how many minutes they spent meditating and practicing yoga, and whether they engaged in any of the mindful living activities, according to their condition. Furthermore, we assessed the subjective experiences participants had during each of these practices. We reported the detailed results regarding these variables in another publication [47]. Qualitative data were obtained from several free text items. In the daily questionnaire, participants could describe any special events that occurred on that day. At 2- and 12-month follow-up, we asked the participants to respond to several open-ended questions to reflect their experiences during and after the course and to describe whether and how they continued to practice the components of their course.

### 2.5. Data Analysis

Data analysis of single-case studies can be manifold [59,60], as we reported in a previous publication [47]. Commonly, the progression of the dependent variables over time are plotted in graphs, which are then visually analyzed [61]. In addition, multiple analysis strategies are chosen to increase the reliability and generalizability of findings. Accordingly, we analyzed data by visual inspection, calculating effect sizes, and multilevel modeling. Moreover, we supplemented our analyses with qualitative findings to explore possible reasons for interindividual differences.

During visual inspection, we assessed trends in the baseline and the treatment phase, differences between the means and the variability of data in each phase, and the consistency of patterns across individuals and conditions. For an experimental single-case study, our sample was exceptionally large. Therefore, we kept our visual analyses relatively simple. Tau-*U* is a family of non-parametric effect size estimates that allow for controlling trends in both phases [62]. We calculated Tau-*U* coefficients for each participant and corrected trends in any phase when they were statistically significant or larger than 0.40. An effect size of less than 0.28 indicated a small effect; 0.29–0.47 a moderate effect; 0.48–0.57 a large effect; and 0.58 or above a very large effect [63].

Multilevel modeling (or hierarchical linear modeling) is a powerful tool for examining both individual change and group differences [64,65]. In this study, changes over time were modeled on one level and differences between individuals on a second level. We standardized all variables for the analyses. Time was coded with zero for the baseline phase and a logarithmic trend starting at the beginning of the treatment phase. We modeled time as a random slope and applied one-tailed tests as we expected all treatments to improve our dependent variables. To assess the proportion of explained variance in each model, we assessed marginal *R*^2^ and conditional *R*^2^ [66]. All models were estimated using the restricted maximum likelihood estimation procedure. 

We examined the incremental effects of the four conditions in an exploratory manner with (1) three dummy variables coding the four conditions (condition model), and (2) two other dummy variables coding the inclusion of the physical yoga or ethical education component (component model). These dummy variables were entered as predictors into two sets of regression models. First, we estimated simple regression models with the Tau-*U* effect sizes as criterion. Second, consistent with the first procedure, we modeled cross-level interactions between time and condition in our multilevel models with the continuous measures of our dependent variables as criterion. Both analyses produce comparable results. All models controlled for individual practice time, age, gender, occupation, and baseline length. Individual practice time was calculated by summing the reported length of each home practice. We hypothesized that combined interventions would have stronger effects than meditation alone and applied one-tailed tests of significance to the dummy variables. We considered *p* < 0.05 to be statistically significant. 

All statistical analyses were performed using R 4.2.0 [67] and the statistical packages *lattice* [68], *ggpubr* [69], *scan* [70], and *nlme* [71]. Proportion of explained variance in multilevel models was calculated using the R-based online application *mimosa* [72]. All scripts and data that support the results can be found at https://osf.io/wnxk6/. This study’s design was preregistered at clinicaltrials.gov under NCT04252976.

## 3. Results

For the three continuously measured variables (body awareness, emotion regulation, affectivity), we report the results of our visual inspection, effect size estimation, and multilevel modeling on the general as well as incremental effects we observed. Then, we explore the relations between these three dependent variables before reporting the results on distress tolerance and self-compassion. Finally, we expand on the long-term effects and observations of specific single cases with possible explanations of these.

### 3.1. Body Awareness

**Figure 1:**
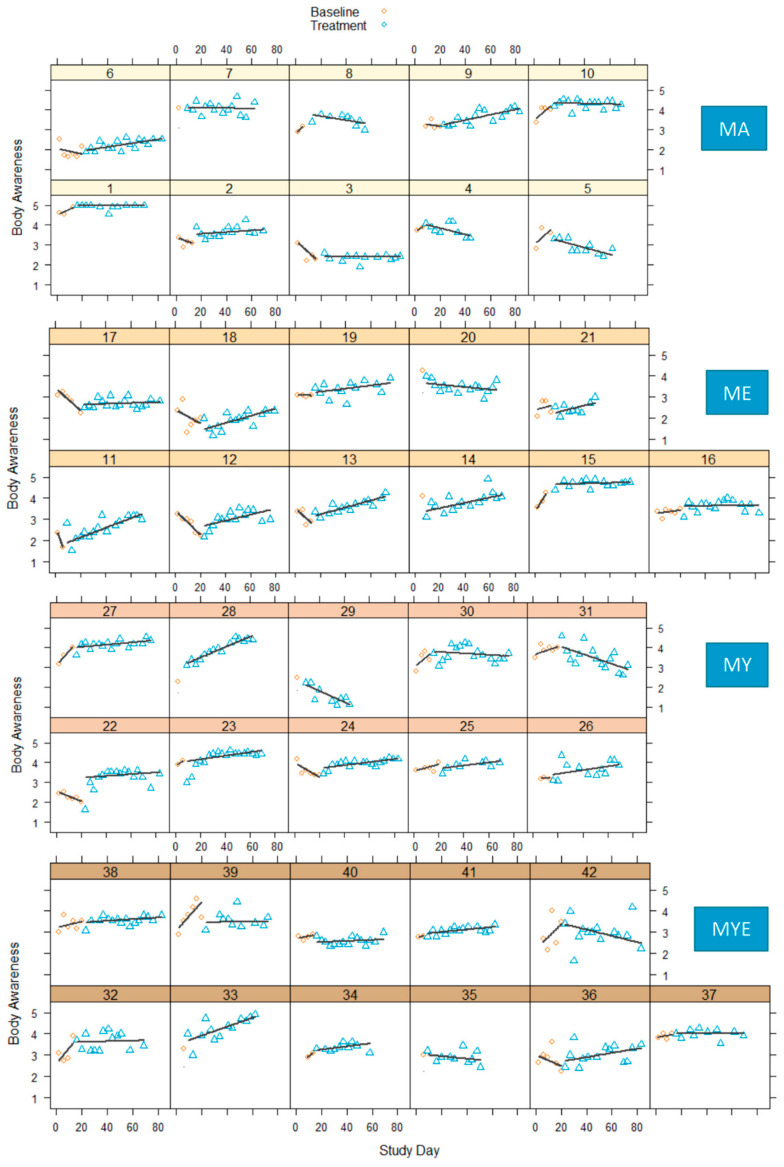
Body awareness scores in four conditions during baseline and treatment phases for each participant with regression lines for each phase. *Note*. MA = Mantra meditation only; ME = meditation and ethical education; MY = meditation and physical yoga; MYE = meditation, physical yoga, and ethical education.

Figure 1 illustrates the development of body awareness scores of each participant over time. Most participants’ body awareness did not significantly fluctuate, indicating that this construct was relatively stable and not subject to daily variation. Overall, there were observable interindividual differences in the general level and slope of the curves. 

During baseline, most participants showed no change or a decrease in body awareness. A few participants’ body awareness increased during baseline. Both increases and decreases in baseline could be due to the heightened sensitivity to the instrument. Nevertheless, the majority of participants showed a steady improvement in body awareness over the course of the treatment with effect sizes ranging from 0.26 to 0.87 (for full tables see Supplementary Material—Table S4). The increase was gradual for most participants starting with the beginning of the treatment or after a small delay. It seems as if being introduced to meditative practices influenced body awareness quite quickly, but continuous practice led to steady improvements. Only two participants showed no observable changes and had effect sizes close to zero. Nine participants experienced a moderate deterioration of body awareness with effect sizes ranging from −0.28 to −0.59. 

Moreover, we examined whether these changes persisted until follow-up two months after the study. Therefore, we compared the mean body awareness scores from phase B (*M_B_* = 3.45, *SD_B_* = 0.71) with the 2-month follow-up measurements (*M_FU2_* = 3.62, *SD_FU2_* = 0.84), and found significant improvements, *t*(39) = 2.58, *p* = 0.014. Interestingly, this did not apply to participants who stopped meditating after the study—their body awareness was generally lower (*M_B_* = 3.01, *SD_B_* = 0.75) and remained at follow-up (*M_FU2_* = 3.06, *SD_FU2_* = 0.99). In addition, we performed an analysis of variance for the 32 participants who completed all three measurements. While this model was not significant, *F*(2, 62) = 2.87, *p* = 0.064, there was a medium effect size, η^2^ = 0.09. This suggests that body awareness improved from post-test (*M_B_* = 3.38, *SD_B_* = 0.77) to 2 months (*M_FU2_* = 3.55, *SD_FU2_* = 0.89) and 12 months after the study (*M_FU12_* = 3.57, *SD_FU12_* = 0.67).

**Figure 2:**
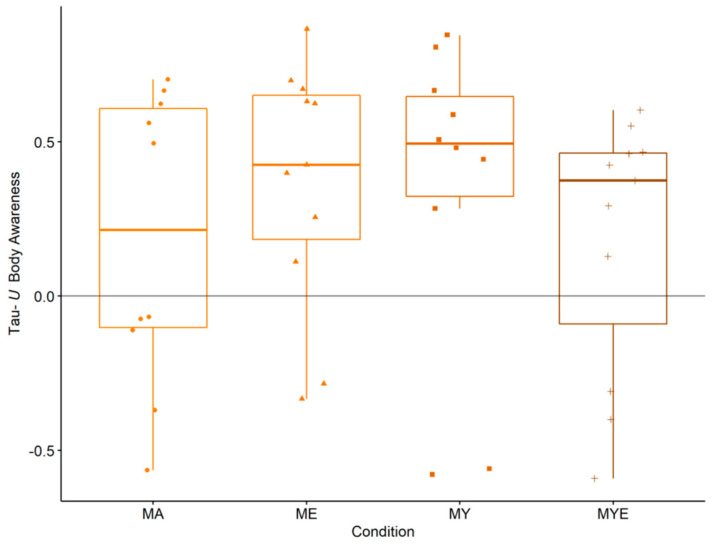
Box plots for averaged Tau-*U* body awareness effect size estimates in each condition. Individual estimates are scattered across the box plots. *Note.* MA = Mantra meditation only (dots); ME = meditation and ethical education (triangles); MY = meditation and physical yoga (squares); MYE = meditation, physical yoga, and ethical education (pluses). Whiskers represent the largest and lowest values within a distance of 1.5 times the interquartile range.

Then, we investigated possible differences between the four conditions. Participants whose body awareness did not change or declined were found in every condition, but particularly in the MA condition. The most improvements were found in the ME and MY conditions. We further explored the incremental effects of our treatments by grouping all Tau-*U* effect size estimates by condition and generating four box plots (Figure 2).

According to Figure 2, all conditions led to an improvement in body awareness with the greatest enhancement in the MY condition (*Mdn* = 0.49, interquartile range [IQR] = 0.32), followed by the ME condition (*Mdn* = 0.43, IQR = 0.47) and the MYE condition (Mdn = 0.38, IQR = 0.32). The MA condition had the smallest effect (*Mdn* = 0.21, IQR = 0.71). When we estimated the total effect of group differences in multiple regression analysis, we found no differences between the conditions, *F*(3, 33) = 0.52, *p* = 0.675. None of the conditions or components nor practice time, age, gender, occupation, or baseline significantly predicted changes in body awareness over time. Multiple *R^2^* was 0.13 and 0.11 for the condition and component model, respectively. In the multilevel model estimating the overall effects, there was a significant effect of time, *F*(1, 677) = 10.23, *p* = 0.001, but no significant cross-level interaction, *F*(3, 677) = 0.47, *p* = 0.702. Both models predicted a significant enhancement of body awareness over time, β = 0.15, *SE* = 0.05, *p* = 0.001 (condition and component model). Nevertheless, there were no other significant effects and no cross-level interactions. The effect sizes for both models were marginal *R^2^* = 0.07 and conditional *R^2^* = 0.86. All regression tables can be found in the Supplementary Material—Tables S5 to S8.

In summary, although there was substantial interindividual variation between participants, all four treatments helped the participants in enhancing their body awareness. This finding is supported by all of our three analyses and is independent of other potentially influential variables. 

### 3.2. Emotion Regulation

**Figure 3:**
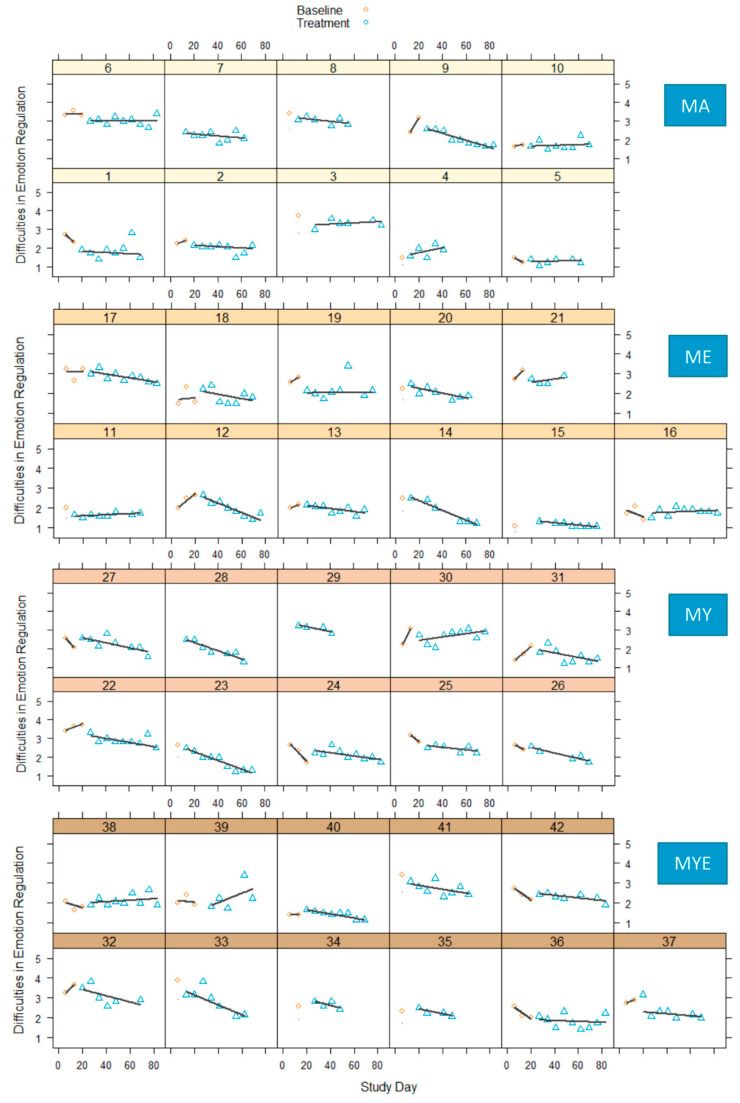
Difficulties in emotion regulation in four conditions during baseline and treatment phases for each participant with regression lines for each phase. *Note*. MA = Mantra meditation only; ME = meditation and ethical education; MY = meditation and physical yoga; MYE = meditation, physical yoga, and ethical education.

Figure 3 displays how the difficulties in emotion regulation developed over time for each participant. Similar to body awareness, emotion regulation did not significantly fluctuate, but slopes and levels varied between participants. It was difficult to make reliable inferences on baseline trends, as there were only few baseline reference points. Three participants had to be excluded from the statistical analysis due to missing baseline data.

When we tentatively compared both phases, we found that most participants exhibited a clear downward trend regarding their difficulties in emotion regulation. This indicates that they improved their ability to be aware of and regulate their feelings. A negative sign in effect sizes represented reduced difficulties in emotion regulation and, thus, a change in the expected direction. Three quarters of the effect size estimates ranged from −0.30 to −0.90 indicating moderate to very large effects (see Supplementary Material—Table S9). For most participants, this amelioration became gradually stronger over time. Very few participants showed no observable changes or increased difficulties in emotion regulation. When we compared the mean scores from phase B (*M_B_* = 2.21, *SD_B_* = 0.52) with the 2-month follow-up measurements (*M_FU2_* = 2.14, *SD_FU2_* = 0.60), we found no significant differences, *t*(37) = −1.16, *p* = 0.255, indicating the absence of a pronounced change over time. This effect remained stable until 12 months later, *F*(2, 62) = 0.19, *p* = 0.827, η^2^ = 0.01. The mean scores for this subgroup were: *M_B_* = 2.23, *SD_B_* = 0.59; *M_FU2_* = 2.18, *SD_FU2_* = 0.64; and *M_FU12_* = 2.21, *SD_FU12_* = 0.70.

**Figure 4:**
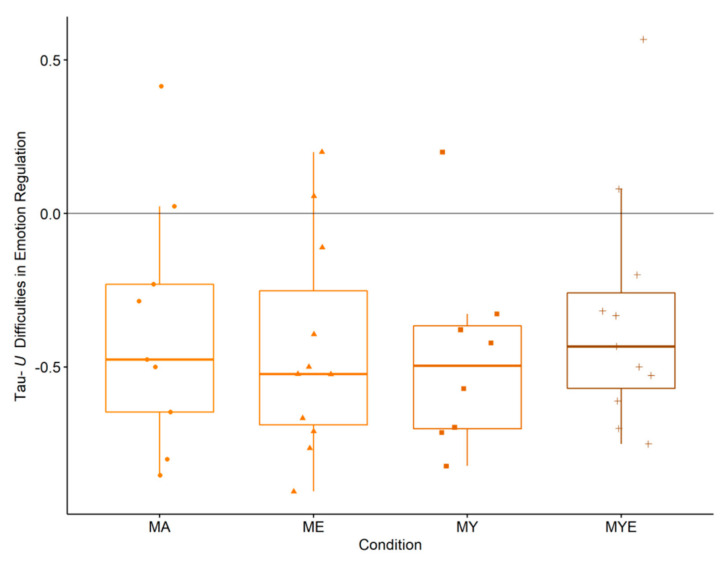
Box plots for averaged Tau-*U* difficulties in emotion regulation effect size estimates in each condition. Individual estimates are scattered across the box plots. *Note*. MA = Mantra meditation only (dots); ME = meditation and ethical education (triangles); MY = meditation and physical yoga (squares); MYE = meditation, physical yoga, and ethical education (pluses). Whiskers represent the largest and lowest values within a distance of 1.5 times the interquartile range.

All conditions comprised participants with large effect sizes, whereas the conditions ME and MY comprised the most. In every condition, we found one participant whose emotion regulation skills weakened over time. Figure 4 depicts box plots with effect size estimates grouped by condition.

Figure 4 demonstrates that all four conditions helped the participants in improving their emotion regulation considerably: ME condition (*Mdn* = −0.52, IQR = 0.44), MY condition (*Mdn* = −0.50, IQR = 0.34), MA condition (*Mdn* = −0.48, IQR = 0.42), and MYE condition (*Mdn* = −0.43, IQR = 0.31). The overall regression estimation indicated no significant between-group effect, *F*(3, 30) = 0.23, *p* = 0.873. None of the conditions/components nor any of the moderators significantly predicted changes in emotion regulation. Multiple *R^2^* was 0.10 and 0.09 for the condition and component model, respectively. In the overall multilevel model, there was an effect of time, *F*(1, 334) = 30.62, *p* < 0.001, but no significant cross-level interaction, *F*(3, 334) = 0.60, *p* = 0.617. Both models predicted a significant improvement in emotion regulation over time, β = −0.26, *SE* = 0.05, *p* < 0.001. Once again, there were no significant predictors nor significant cross-level interactions, suggesting that all conditions enhanced regulatory skills equally well. Effect sizes for both models were marginal *R^2^* = 0.19 and conditional *R^2^* = 0.81. For full regression tables, see Supplementary Material—Tables S10–S13. Summarizing the three analyses, all four treatments strengthened participants’ abilities to regulate their emotional responses. 

### 3.3. Affectivity

**Figure 5:**
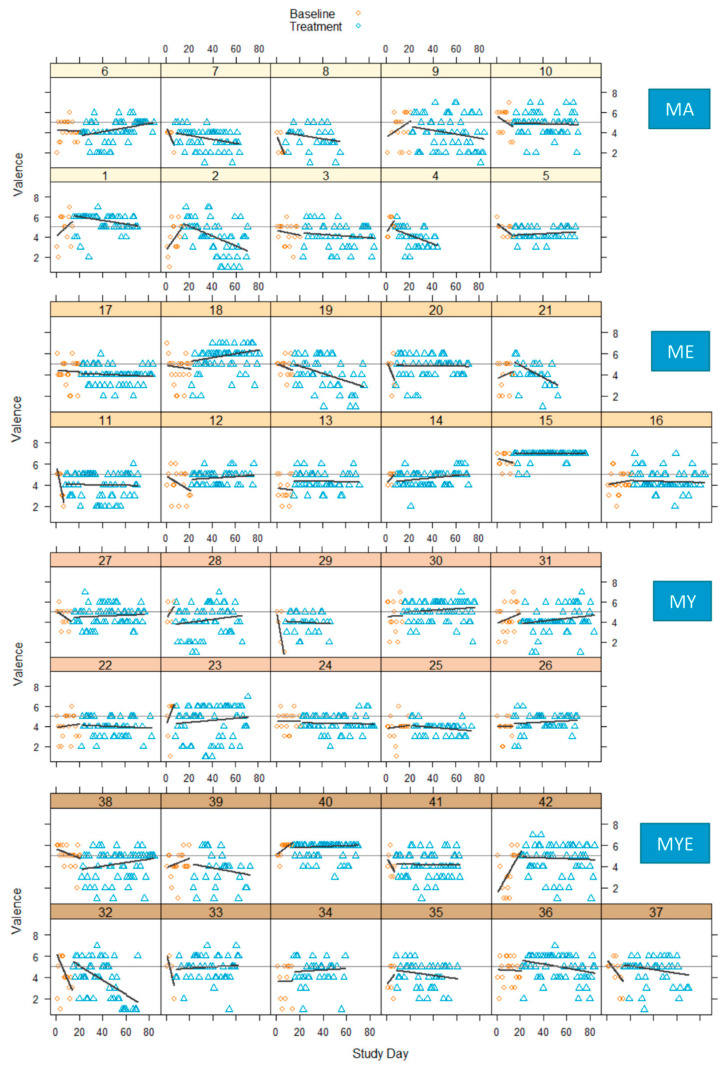
Valence scores in four conditions during baseline and treatment phases for each participant with regression lines for each phase. *Note*. Thin horizontal line represents a neutral valence (value = 5); MA = mantra meditation only; ME = meditation and ethical education; MY = meditation and physical yoga; MYE = meditation, physical yoga, and ethical education.

**Figure 6:**
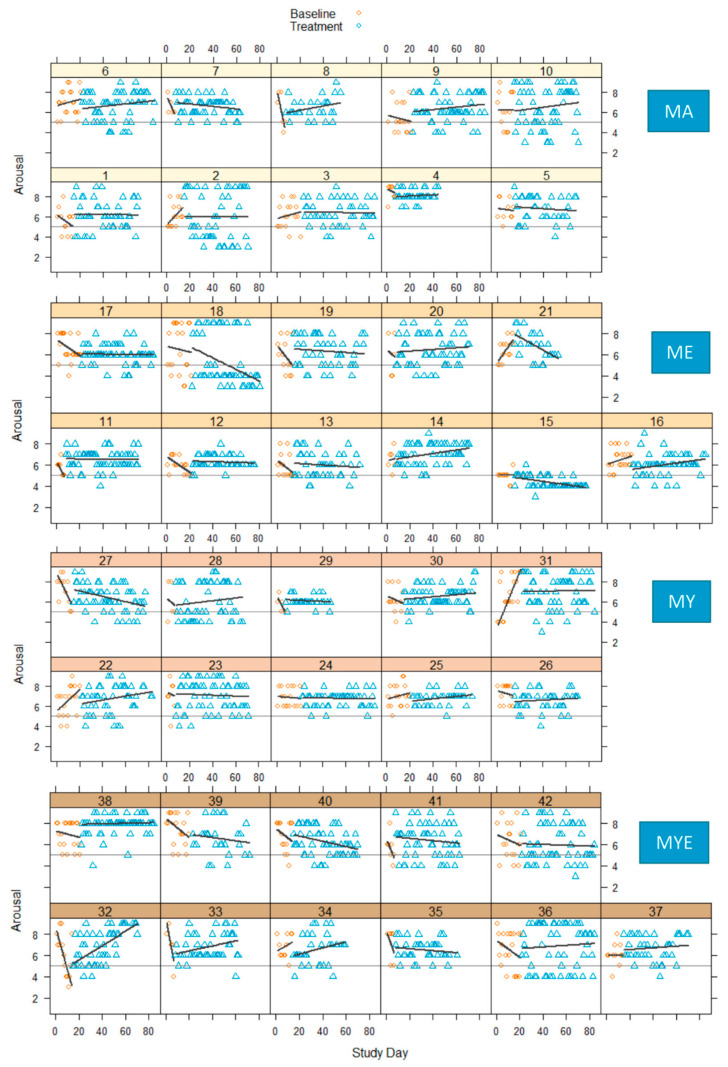
Arousal scores in four conditions during baseline and treatment phases for each participant with regression lines for each phase. *Note*. Thin horizontal line represents a neutral arousal (value = 5); MA = mantra meditation only; ME = meditation and ethical education; MY = meditation and physical yoga; MYE = meditation, physical yoga, and ethical education.

The variable-by-time plots for valence and arousal are depicted in Figure 5 and Figure 6, respectively. A thin horizontal line was drawn into each graph at the score 5 to delineate a neutral valence or arousal that is neither high/positive nor low/negative [55]. 

**Figure 7:**
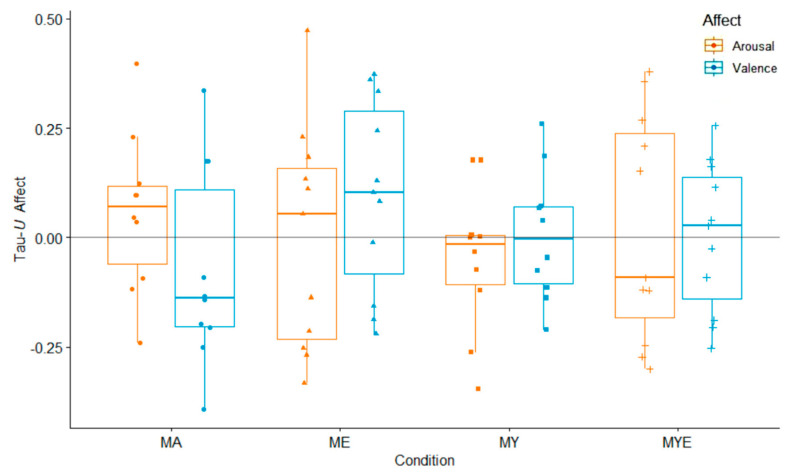
Box plots for averaged Tau-*U* arousal and valence estimates in each condition. Individual arousal and valence estimates are scattered across the box plots. *Note*. MA = Mantra meditation only (dots); ME = meditation and ethical education (triangles); MY = meditation and physical yoga (squares); MYE = meditation, physical yoga, and ethical education (pluses). Whiskers represent the largest and lowest values within a distance of 1.5 times the interquartile range.

The visual inspection of these plots indicated strong inter- and intra-individual fluctuations in both affective dimensions over time. In most cases, the baseline phase displayed a stronger upward or downward trend than the treatment phase. These trends reversed or leveled out during the treatment phase. The variability of scores was comparable across both phases. Effects were inconsistent across individuals. Both arousal and valence increased, decreased, or stayed the same from baseline to treatment. A majority of effect size estimates ranged from −0.25 to 0.37 for valence, and −0.30 to 0.40 for arousal, indicating a great variability in the results (see Supplementary Material—Table S14). Valence declined for a majority of participants from the MA condition, but improved for many participants from the ME condition. Figure 7 displays the grouped effect sizes of all four treatments on both variables.

The average effect sizes for arousal were close to zero and did not differ significantly between conditions—MA (*Mdn* = 0.07, IQR = 0.18), ME (*Mdn* = 0.05, IQR = 0.39), MY (*Mdn* = −0.02, IQR = 0.11), and MYE (*Mdn* = −0.09, IQR = 0.42). However, there was a discernible difference in valence effect sizes between the conditions MA (*Mdn* = −0.14, IQR = 0.31) and ME (*Mdn* = 0.10, IQR = 0.37). The average effect size for valence in the other two conditions was close to zero—MY (*Mdn* = −0.00, IQR = 0.18) and MYE (*Mdn* = 0.03, IQR = 0.28). We had to exclude one participant from the regression analyses of arousal as she was an outlier who was considerably older and had substantially elevated levels of arousal. The aggregated effect of all four conditions for valence and arousal was *F*(3, 33) = 1.17, *p* = 0.334 and *F*(3, 32) = 0.73, *p* = 0.543, respectively, suggesting no relevant differences between conditions. In the condition model of valence, receiving the ME treatment significantly predicted an increase in valence over time, β = 0.41, *p* = 0.041, suggesting that this condition improved positive affect. Neither ethical education or physical yoga nor any other variable significantly predicted changes in valence and arousal. However, in the condition model of arousal, being employed (compared to being a student) was a significant predictor of increased arousal over time, β = 0.45, *p* = 0.038. Multiple *R*^2^ was 0.12 and 0.07 for valence (condition/component model), and 0.27 and 0.22 for arousal.

In the overall multilevel model, we found no interaction effect for valence or arousal, *F*(3, 2492) = 1.89, *p* = 0.129, and *F*(3, 2454) = 0.00, *p* = 0.708, respectively. There were no significant predictors in the arousal models. In the condition model of valence, there were two significant cross-level interactions between time and the ME condition, β = 0.08, *SE* = 0.03, *p* = 0.013, and time and the MY condition, β = 0.06, *SE* = 0.03, *p* = 0.033, indicating an increase in valence over time. Neither the yoga nor the ethical component predicted valence in the component model. Therefore, the positive effect was limited to the two conditions that involved meditation, in addition to one additional component, but did not depend on a specific component. Interestingly, the only (other) significant predictor of valence in both models was the total practice time that the individuals completed, β = 0.17, *SE* = 0.08, *p* = 0.033/0.031. Therefore, the more participants engaged in a regular home practice, the more their valence increased. This was independent of their specific condition, although total practice time was longer in the more extensive conditions. The effect sizes for the final component models for valence/arousal were marginal *R^2^* = 0.04/*R^2^* = 0.04 and conditional *R^2^* = 0.22/*R^2^* = 0.15. Full regression tables can be found in the Supplementary Material—Tables S15–S22.

**Figure 8:**
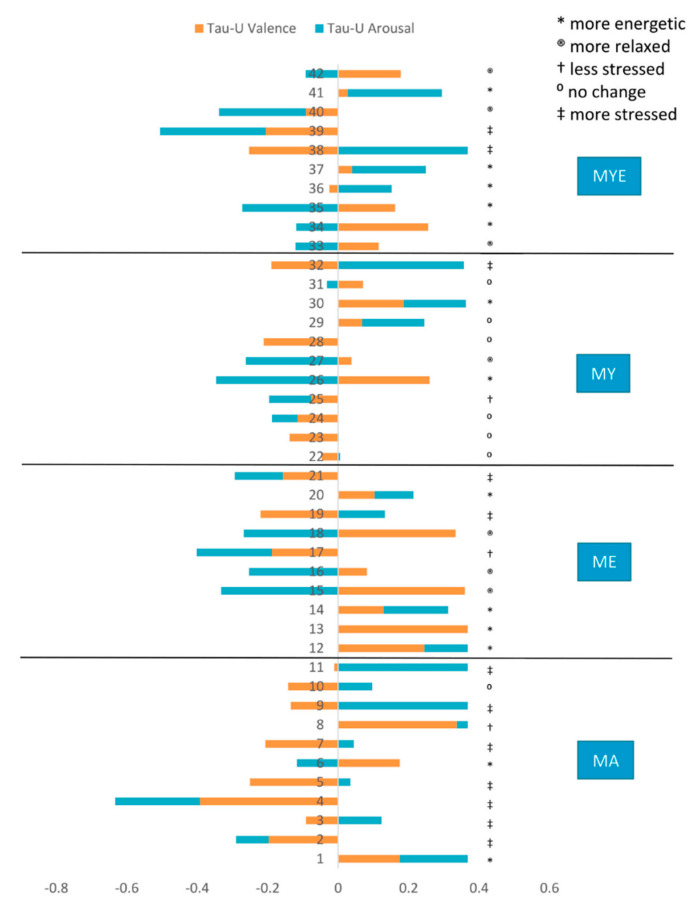
Stacked bar chart of Tau-*U* valence and arousal estimates with qualitatively classified affective responses per participant. *Note*. Numbers denote participants; MA = mantra meditation only; ME = meditation and ethical education; MY = meditation and physical yoga; MYE = meditation, physical yoga, and ethical education.

Next, we considered both dimensions simultaneously, visually and by placing effect sizes of both variables side by side in a combined figure (Figure 8). As a result, we developed five main categories of affective responses to our treatment, which are depicted with different symbols in Figure 8. The majority of participants experienced a positive change in affect, a small group of participants showed no observable changes, and some participants felt more stressed. 

Next, we compared the frequencies of each category of affective response in each condition (see Supplementary Material—Table S23). Around two-thirds of participants in the MA condition felt more stressed over the course of time. Half of the participants from the MY condition experienced no change in their affect. However, no one from this group was classified as being more stressed, suggesting a stress-relieving effect of physical yoga. Participants from both groups that received ethical education (ME and MYE) were more energetic, more relaxed, or more stressed during the intervention. Therefore, the ethical education component elicited multi-layered responses. This contrasts and complements our previous analyses where the MY and specifically the ME condition had a favorable effect on valence, whereas the MA condition was less advantageous.

### 3.4. Relations between Affectivity, Body Awareness, and Emotion Regulation

Research literature postulates a close connection between body awareness and emotion regulation [30]. Therefore, we explored this connection by correlating effect size estimates of both variables. We found a moderate correlation (*r* = −0.24), indicating that when participants’ body awareness increased, their difficulties in emotion regulation decreased, as well. We found similar patterns and trajectories for both variables during visual inspection, indicating that both skills develop simultaneously during contemplative training.

Next, we identified whether there were any differences between the conditions regarding this relation. Interestingly, correlations were significantly higher in the MA (*r* = −0.53) and MY (*r* = −0.43) conditions compared to both conditions that received ethical education: ME (*r* = 0.20) and MYE (*r* = −0.15). Therefore, in treatments where participants were mainly focused on observing rather than actively changing themselves, increases in body awareness were more strongly related to increases in emotion regulation. 

Furthermore, we were interested in how changes in both skills were related to changes in daily affect. We found that decreases in difficulties in emotion regulation were higher when the affective response was classified as positive (*Mdn* = −0.52, IQR = 0.31), compared to no change (*Mdn* = −0.38, IQR = 0.37) or negative (*Mdn* = −0.29, IQR = 0.66). In contrast, enhancements in body awareness were highest when participants experienced no change in affectivity (*Mdn* = 0.59, IQR = 0.79), compared to positive (*Mdn* = 0.43, IQR = 0.36) or negative (*Mdn* = 0.24, IQR = 0.75). This indicates that emotion regulation and affective experience co-varied, whereas body awareness and affective experience did not. 

### 3.5. Distress Tolerance

For distress tolerance, a mixed two-way ANOVA showed a significant effect of time, *F*(2, 72) = 23.01, *p* < 0.001, with a large effect size, η^2^ = 0.13. There were no significant differences between the four conditions, *F*(3, 36) = 1.27, *p* = 0.299, nor a significant interaction between time and condition, *F(*6, 72) = 0.51, *p* = 0.746. The mean distress tolerance across all conditions was *M_pre_* = 3.42 (*SD* = 0.74) before the study, *M_post_* = 3.95 (*SD* = 0.72) at completion of the study, and *M_fu_* = 3.99 (*SD* = 0.70) 2 months after the study. Therefore, all treatments markedly enhanced distress tolerance from pre- to post-test, which was sustained until 2-month follow-up. 

### 3.6. Self-Compassion

Another mixed two-way ANOVA yielded a significant effect of time for self-compassion, *F*(1, 37) = 26.25, *p* < 0.001, with a medium effect size, η^2^ = 0.08. Once again, there were no significant differences between the four conditions, *F*(3, 37) = 1.23, *p* = 0.311, nor a significant interaction between time and condition, *F*(3, 37) = 1.57, *p* = 0.213. The mean self-compassion across all conditions increased from *M_pre_* = 3.00 (*SD* = 0.60) before the study to *M_post_* = 3.37 (*SD* = 0.64) at completion of the study. Accordingly, all treatments helped the participants in cultivating their ability to be kind and forgiving to themselves.

### 3.7. Changes in Home Practice

The reported long-term effects were found although the practice times declined considerably from the last month of the treatment to 2- and 12-month follow-up (Table 1). We calculated the accumulated minutes of each practice per month to obtain comparable values. The engagement in all practices decreased over time, but meditation and ethical practice decreased more steeply than the yoga practice. Looking at the reported practice times at 12-month follow-up, we found another interesting result. Ethical practice was reported mainly in the two conditions that included ethical education. Meditation practice times were comparable across conditions. However, surprisingly, the physical yoga practice was reported consistently in all four conditions, with the highest mean practice time in the MYE condition (3 h per month). Similarly, of all 33 participants who completed the 12-month follow-up, 63.4% still meditated, 57.6% practiced ethics, and 72.7% practiced yoga.

### 3.8. Selected Cases

Subsequently, we looked at potential explanations for the negative effects we found for body awareness and emotion regulation. Participants 5, 29, 31, and 35 experienced a considerable decline in body awareness throughout the treatment. Participant 5 (MA) was classified as becoming more stressed throughout the treatment, but markedly improved his distress tolerance over time. At 12-month follow-up, he stated being very grateful for the course, as he had learned a valuable relaxation technique that he could rely on, especially when things were getting stressful. He had meditated regularly during the course and had continued to meditate until the present time. We had considered excluding Participant 29 (MY) as she was not very conscientious in responding to our daily questionnaires and stopped responding by Week 5 of the treatment. Nevertheless, she regularly attended her class although she did not seem very enthusiastic during class and did not practice at home very often neither during the study nor afterwards. Interestingly, her affective experience did not change, her self-compassion declined, but her distress tolerance improved. On the other hand, participant 31 (MY) was very enthusiastic about her course, even in retrospective, and reported that particularly yoga became part of her daily routine up to the present time. She had high levels of distress tolerance from the beginning, her affective experience did not change, and her self-compassion increased over the course of the study. Participant 35 (MYE) conscientiously practiced yoga and meditation during the study, but lost motivation to continue although he was still interested. He found working out and being in nature more helpful. Throughout the study, he became more energetic, but his self-compassion or distress tolerance did not change significantly. In summary, although all four participants experienced a decline in body awareness, they responded quite differently (and often positively) to the treatment in other variables. 

Only two participants reported increased difficulties in emotion regulation over time. Interestingly, Participant 4 (MA) was the only participant who exhibited a deterioration in all three continuous dependent variables (affect, body awareness, emotion regulation). Nevertheless, her distress tolerance improved. Participant 38 (MYE) was classified as becoming more stressed and her distress tolerance declined, but her body awareness and self-compassion increased. We identified and discussed these two participants in another publication as they also experienced a considerable decline in well-being [47]. Indeed, effect sizes of difficulties in emotion regulation and well-being were highly correlated (*r* = −0.41). Nevertheless, both participants retrospectively appreciated their course and continued to meditate regularly. This might be an indication that the treatment led to an initial worsening during the process of change.

## 4. Discussion

The present study investigated the specific and incremental benefits of adding physical Hatha yoga and ethical education to a mantra meditation intervention in healthy participants. It evaluated processes related to embodied cognition in four different combinations of yoga components, which are part of the new mind–body therapy MBLM [44]. The single-case multiple-baseline design made it possible to evaluate individual responses and change processes over time. All four treatments had positive effects on body awareness, emotion regulation skills, distress tolerance, and self-compassion. Effects were strongest for emotion regulation and distress tolerance. Surprisingly, there were no significant between-group differences regarding these variables although the treatments differed considerably in session duration and complexity. In contrast, findings on daily affect were more differentiated, but also less conclusive. The MA condition had the least favorable effect on valence and overall affectivity, whereas the ME condition improved valence the most and the MY condition elicited no negative change in affectivity. However, due to the exploratory nature of our study, and the relatively small sample size, our findings should be regarded as preliminary.

Nonetheless, these results complement and extend our previously reported findings on well-being and stress [47]. In this earlier publication, we elucidated that the simple meditation treatment was the least effective and led to increased stress and decreased well-being in a sizeable number of participants. Furthermore, the two less extensive combinations of yoga components, namely, the ME and MY condition were the most helpful in enhancing well-being (ME) and reducing stress (MY). This is in line with the abovementioned results on affectivity. Nevertheless, the condition-independent positive effects on the other dependent variables seem somewhat puzzling. These indicate that factors common to all four interventions were responsible for the observed changes in these variables. These factors might be unspecific, such as social support, group dynamics, or attention from the intervention teachers [41,73]. On the contrary, most effects persisted until follow-up, some even until 12 months after the intervention had ended. Therefore, it seems more plausible to assume that these effects were caused by the specific shared component of mantra meditation. 

### 4.1. Mantra Meditation Might Be the Driving Force behind Subjective Improvements 

Previous studies have substantiated the positive effects of mantra meditation on mental health and affectivity in healthy populations, although methodological flaws limit the validity of these findings [74,75]. Our study provides methodologically sound evidence for the positive effects of mantra meditation on body awareness, emotion regulation, self-compassion, and distress tolerance, but not necessarily on positive affect. Interestingly, the former variables were neither influenced by differences in session duration nor by the fact that participants were fully disclosed about the study design. Therefore, mantra meditation seems to have had a quite powerful impact. Fittingly, meditation is regarded as the key element in classical yoga (ashtanga yoga) as outlined by Patanjali, with ethics, postures, and breathing practices being considered as pre-requisites or preparatory exercises [76]. 

Schmalzl et al. [9] proposed that meditation leads to an increase in interoception and metacognitive awareness. Accordingly, interoception has been suggested as one of the central mechanisms of meditation [77,78]. Furthermore, neuroscientific studies have shown consistent activations in brain regions associated with interoceptive processes (e.g., insula) across many different kinds of meditation [79]. In a recent comparative study, walking meditation, humming meditation, concentration on an object, and observing-thoughts meditation led to similar increases in body awareness and emotion regulation in novice meditators [80]. According to a recent classification system, mantra meditation does not have a strong orientation toward the body [81]. Nevertheless, it encourages meditators to turn their attention inwards, which might explain why it enhanced the processing of bodily and emotional stimuli in the present study. Similarly, the sustained focus on a simple, repetitive stimulus (mantra) resembles states of sensory deprivation and perceptual isolation [82]. These states have been shown to intensify or alter sensory perceptions and make them more salient [83]. Additionally, participants were instructed to be kind and friendly toward themselves and their experiences during meditation. This might have enhanced their self-compassion and tolerance of negative emotions [84]. Consequently, the strong focus of meditation on internal processes and on increasing self-awareness might be a central mechanism underlying the observed changes.

### 4.2. Adding Yoga Components to Meditation Enhances Positive Affect

In contrast, mantra meditation alone exhibited a partly negative effect on participants’ daily affect. In this respect, adding other yoga components to the treatment proved to be a valuable extension and possibly provided participants with additional inspiration and a frame of reference [47]. While there were no significant between-group differences regarding body awareness, effect sizes were slightly higher in the groups with additional yoga components. Remarkably, it mostly did not matter which component was added or practiced in this regard. Physical Hatha yoga emphasizes becoming aware of the (moving) body and has been found to increase vagal/parasympathetic activity [8,25]. This might explain its balancing effect on affectivity. On the other hand, ethical education encourages the development of value-oriented behavior [1], and is associated with improved well-being, self-compassion, and anxiety [85,86]. This is in accordance with its positive effects on valence as reported above. Intriguingly, physical yoga was the only component that was consistently practiced until 12-month follow-up, even in conditions that did not originally include physical yoga practice. This indicates that physical yoga might be easier to integrate into participants’ lives, corresponding with earlier findings [87].

### 4.3. Body Awareness and Emotion Regulation Are Central Mechanisms

Body awareness and emotion regulation developed steadily and continuously over time, whereas daily affect fluctuated significantly. This indicates that the former are important skills and possible mechanisms of action that develop simultaneously over time. Both skills have been described as central to meditation [83], but more research is warranted to substantiate our findings. In addition, we found stronger correlations between changes in both skills in the non-ethical than in the ethical conditions. This supports the notion that postures, breathing, and meditation encourage an integrated and embodied way of processing and modulating bodily and emotional responses [8]. On the other hand, the ethical education component might have strengthened top-down processing and, thus, a decoupling of body awareness and emotion regulation. This corresponds to the finding that an (embodied) yoga intervention was more effective than a (non-embodied) cognitive-behavioral stress-management course in enhancing interoceptive awareness and emotion regulation [35].

We observed substantial interindividual variation in response to the treatments. While the majority of participants benefitted from the treatments, a few participants became more stressed or experienced a decline in their body awareness and/or emotion regulatory skills. As we know from psychotherapeutic contexts, change is not always easy and often leads to an initial impairment of symptoms [88]. This might have happened to some of our participants, as well, since most of them were quite grateful for their course 12 months after the study. Moreover, a decrease in body awareness might indicate a shift in participants’ frame of reference. Specifically, participants might be more aware or more mindful toward their inner experience during the treatment and, thus, adjust their responses [89,90]. Similarly, individual characteristics might influence a participant’s response to the different yoga components [9,48,84]. 

## 5. Limitations and Future Directions

Although our study employed rigorous methodology, it had a few limitations, as well. First, participants in our study were presumably intrinsically motivated to participate in this study. Second, the intense data gathering was very demanding for participants and led to a relatively high number of dropouts and missing data. Therefore, providing financial or other compensation might help in enhancing participant commitment. Third, our sample size was somewhat limited for certain aspects of the evaluation. A sample of 42 participants is considered exceptionally large for an experimental single-case study. The large number of measurements (up to 85 for daily measures) makes our findings and effect size estimates very reliable. This applies less to measures collected less often and to between-group comparisons. However, a post-hoc power analysis indicated that our study was well-powered to perform pre-post-follow-up analyses with medium to large effects. For the continuous measures, we performed both regression analyses and multilevel modeling to increase the reliability our findings. Finally, our results are hypotheses generating rather than testing. Therefore, they could guide future research with preferably larger subgroups. 

Furthermore, dependent variables should be collected more frequently with short, specific, and reliable instruments. In the meantime, researchers have developed short forms for measuring emotion regulation [91] and self-compassion [92]. However, our body awareness questionnaire needs to be thoroughly validated to substantiate its usefulness beyond the present study. Daily affect could be assessed more specifically with simple questions, such as “To what extent did you feel happy/sad/calm/stressed today?” Moreover, specific emotion regulatory strategies, including the proposed mindful emotion regulation [33], or specific embodiment processes could be assessed in more detail. In addition, experience-sampling methods [93] represent an alternative research design, which is highly suited for capturing immediate experiences over the course of the day. 

Interestingly, the meditation-only condition, which served as an appropriate control group, produced the same effects for most variables as the more extensive conditions. Future studies could evaluate whether these effects generalize to different types of meditation [81]. Similarly, future studies could match conditions by session length and dismantle the effects of yoga components even further by comparing only ethics, only physical postures, only breathing techniques, or only meditation to diverse combinations of these. Likewise, they could include an active non-yoga and non-meditation (placebo) control group (see, e.g., [94]) to expand on the specificity of the found effects. Furthermore, future studies should investigate how personality and motivational factors impact the outcomes of different treatments to enable personalized recommendations [48,95]. In summary, all of these research efforts could help in developing a deeper understanding of the working mechanisms of the multifaceted practice of yoga and how it contributes to general well-being and flourishing.

## 6. Conclusions

This experimental single-case study provided valuable insights into the working mechanisms of different yoga components. Although the investigated treatments differed in session length and components taught, they had a similarly positive impact on body awareness, emotion regulation, distress tolerance, and self-compassion. This points to the central role of the shared component of mantra meditation in enhancing the processing of bodily and emotional stimuli. Nevertheless, adding other yoga components had a favorable effect on daily affectivity of the participants. Most changes persisted until follow-up, even though participants did not continue to practice. More research is needed to substantiate our findings.

## Figures and Tables

**Table 1:** Accumulated monthly practice of meditation, physical yoga, and ethical practice in minutes for the last month of the treatment, the 2- and 12-month follow-up, and the 12-month follow-up in each condition.

			Meditation	Physical Yoga	Ethical Practice
		*n*	*Mdn* (IQR)	*Mdn* (IQR)	*Mdn* (IQR)
Last month of treatment		41	248 (231)	230 (159)	280 (160)
2-month follow-up		40	80 (160)	100 (343)	40 (80)
12-month follow-up		33	30 (40)	80 (180)	10 (60)
	MA	9	30 (40)	80 (150)	0 (40)
	MY	7	40 (35)	70 (188)	0 (10)
	ME	8	30 (150)	60 (225)	130 (275)
	MYE	9	20 (40)	180 (150)	20 (60)

## Data Availability

Data, analysis, and additional materials are openly available at the Open Science Framework (https://osf.io/wnxk6/).

## Supplementary Materials

The following supporting information can be downloaded at: https://www.mdpi.com/article/10.3390/ijerph191811734/s1, Table S1: Body Awareness Questionnaire Employed in the Present Study With Questionnaires They Were Derived From; Table S2: Shortened Difficulties in Emotion Regulation Scale Employed in the Present Study; Table S3: Shortened Self-Compassion Scale Employed in the Present Study; Table S4: Mean Body Awareness Scores With Standard Deviations for Baseline (A) and Treatment (B) Phases, and Tau-U Estimates With Respective Significance Levels and Type of Tau-U for Each Participant; Table S5: Regression Model for Tau-U Body Awareness Estimates as Dependent Variable and Condition, Total Practice Time, Age, Gender, Occupation, and Baseline Length as Predictors; Table S6: Regression Model for Tau-U Body Awareness Estimates as Dependent Variable and Component, Total Practice Time, Age, Gender, Occupation, and Baseline Length as Predictors; Table S7: Multilevel Regression Estimates for Body Awareness Scores as Dependent Variable and Time, Condition, Total Practice Time, Age, Gender, Occupation, and Baseline Length as Predictors; Table S8: Multilevel Regression Estimates for Body Awareness Scores as Dependent Variable and Time, Component, Total Practice Time, Age, Gender, Occupation, and Baseline Length as Predictors; Table S9: Mean Difficulties with Emotion Regulation Scores With Standard Deviations for Baseline (A) and Treatment (B) Phases, and Tau-U Estimates With Respective Significance Levels and Type of Tau-U for Each Participant; Table S10: Regression Model for Tau-U Difficulties with Emotion Regulation Estimates as Dependent Variable and Condition, Total Practice Time, Age, Gender, Occupation, and Baseline Length as Predictors; Table S11: Regression Model for Tau-U Difficulties with Emotion Regulation Estimates as Dependent Variable and Component, Total Practice Time, Age, Gender, Occupation, and Baseline Length as Predictors; Table S12: Multilevel Regression Estimates for Difficulties with Emotion Regulation Scores as Dependent Variable and Time, Condition, Total Practice Time, Age, Gender, Occupation, and Baseline Length as Predictors; Table S13: Multilevel Regression Estimates for Difficulties with Emotion Regulation Scores as Dependent Variable and Time, Component, Total Practice Time, Age, Gender, Occupation, and Baseline Length as Predictors; Table S14: Mean Valence and Arousal Scores With Standard Deviations for Baseline (A) and Treatment (B) Phases, and Tau-U Estimates and Categorized Affective Response for Each Participant; Table S15: Regression Model for Tau-U Valence Estimates as Dependent Variable and Condition, Total Practice Time, Age, Gender, Occupation, and Baseline Length as Predictors; Table S16: Regression Model for Tau-U Valence Estimates as Dependent Variable and Component, Total Practice Time, Age, Gender, Occupation, and Baseline Length as Predictors; Table S17: Multilevel Regression Estimates for Valence Scores as Dependent Variable and Time, Condition, Total Practice Time, Age, Gender, Occupation, and Baseline Length as Predictors; Table S18: Multilevel Regression Estimates for Valence Scores as Dependent Variable and Time, Component, Total Practice Time, Age, Gender, Occupation, and Baseline Length as Predictors; Table S19: Regression Model for Tau-U Arousal Estimates as Dependent Variable and Condition, Total Practice Time, Age, Gender, Occupation, and Baseline Length as Predictors; Table S20: Regression Model for Tau-U Arousal Estimates as Dependent Variable and Component, Total Practice Time, Age, Gender, Occupation, and Baseline Length as Predictors; Table S21: Multilevel Regression Estimates for Arousal Scores as Dependent Variable and Time, Condition, Total Practice Time, Age, Gender, Occupation, and Baseline Length as Predictors; Table S22: Multilevel Regression Estimates for Arousal Scores as Dependent Variable and Time, Component, Total Practice Time, Age, Gender, Occupation, and Baseline Length as Predictors; Table S23: Frequencies of Categorized Affective Reponses to the Treatment in Each Condition.
