# Supplementary material for: Embodied Cognition in Meditation, Yoga, and Ethics—An Experimental Single-Case Study on the Differential Effects of Four Mind–Body Treatments
Source: Int J Environ Res Public Health. 2022 Sep 17;19(18):11734. doi: 10.3390/ijerph191811734 (PMC9517053; doi:10.3390/ijerph191811734)
Supplement: Supplementary file 1 [file ijerph-19-11734-s001.zip › ijerph-1878494-supplementary.pdf]

Supplementary Material

Embodied Cognition in Meditation, Yoga, and Ethics

An Experimental Single-Case Study on Differential Effects of Four Treatments

Matko, Sedlmeier, & Bringmann (2022)

Table S1

Body Awareness Questionnaire Employed in the Present Study With Questionnaires They Were Derived From

1. I was aware of a cycle in my activity level throughout the day. (BAQ)
2. I knew immediately when my mouth or throat got dry. (PBCS)
3. I noticed changes in my breathing, such as whether it slowed down or sped up. (MAIA)
4. I noticed where in my body I was comfortable. (MAIA)
5. I was sensitive of internal bodily tensions. (PBCS)
6. I felt my heart beating. (PBCS)
7. I was quick to sense the hunger contractions of my stomach. (PBCS)
8. I was aware of changes in my body temperature. (PBSC)
9. I noticed differences in the way my body reacted to various foods. (BAQ)
10. I noticed how my body changed when I was feeling emotions. (MAIA)
11. When I was walking, I deliberately noticed the sensations of my body moving. (FFMQ)

Note. BAQ = Body Awareness Questionnaire; FFMQ = Five Facets of Mindfulness Questionnaire; MAIA = Multidimensional Assessment of Interoceptive Awareness; PBSC = Private Body Consciousness Scale.

Table S2

Shortened Difficulties in Emotion Regulation Scale Employed in the Present Study

1. When I'm upset, I feel guilty for feeling that way.
2. When I'm upset, I feel ashamed with myself for feeling that way.
3. When I'm upset, I have difficulty concentrating.
4. When I'm upset, I have difficulty focusing on other things.
5. When I'm upset, I lose control over my behaviors.
6. When I'm upset, I have difficulty controlling my behaviors.
7. I am attentive to my feelings. (r)
8. I pay attention to how I feel. (r)
9. When I'm upset, I believe that I'll end up feeling very depressed.
10. When I'm upset, I believe that I will remain that way for a long time.
11. I have difficulty making sense out of my feelings.
12. I have no idea how I am feeling.

Table S3

Shortened Self-Compassion Scale Employed in the Present Study

1. When I'm going through a very hard time, I give myself the caring and tenderness I need.
2. I'm kind to myself when I'm experiencing suffering.
3. When I see aspects of myself that I don't like, I get down on myself.
4. I can be a bit cold-hearted towards myself when I'm experiencing suffering.
5. When I feel inadequate in some way, I try to remind myself that feelings of inadequacy are shared by most people.
6. I try to see my failings as part of the human condition.
7. When I fail at something that's important to me I tend to feel alone in my failure.
8. When I think about my inadequacies it tends to make me feel more separate and cut off from the rest of the world.
9. When I fail at something important to me I try to keep things in perspective.
10. When something painful happens I try to take a balanced view of the situation.
11. When I'm feeling down I tend to obsess and fixate on everything that's wrong.
12. When I fail at something important to me I become consumed by feelings of inadequacy.

Table S4

*Mean Body Awareness Scores With Standard Deviations for Baseline (A) and Treatment (B) Phases, and Tau-*U *Estimates With Respective Significance Levels and Type of Tau-*U *for Each Participant*

Case	*Mean* A	*Mean* B	*SD* A	*SD* B	Tau-*U*	Tau-*U* Significance	Tau-*U* Type
1	4.70	4.93	0.19	0.14	**0.667**	.045	A vs. B - Trend A
2	3.14	3.68	0.19	0.25	**0.562**	.001	A vs. B - Trend A + Trend B
3	2.53	2.36	0.41	0.18	-0.111	.719	A vs. B - Trend A
4	3.82	3.79	0.13	0.32	-0.370	.114	A vs. B + Trend B
5	3.44	2.90	0.54	0.34	**-0.564**	.007	A vs. B - Trend A + Trend B
6	1.92	2.26	0.37	0.25	**0.496**	.001	A vs. B + Trend B
7	4.11	4.09	n.a.	0.30	-0.067	.717	A vs. B + Trend B
8	3.05	3.50	0.19	0.27	-0.074	.753	A vs. B + Trend B
9	3.25	3.67	0.20	0.35	**0.624**	.000	A vs. B + Trend B
10	3.89	4.30	0.35	0.21	**0.703**	.033	A vs. B
11	2.03	2.63	0.47	0.48	**0.632**	.000	A vs. B + Trend B
12	2.80	3.02	0.40	0.39	**0.426**	.008	A vs. B - Trend A + Trend B
13	3.11	3.63	0.35	0.31	**0.699**	.000	A vs. B - Trend A + Trend B
14	4.09	3.84	n.a.	0.44	**0.400**	.029	A vs. B + Trend B
15	3.89	4.69	0.34	0.16	**0.867**	.014	A vs. B - Trend A
16	3.33	3.63	0.18	0.25	**0.625**	.027	A vs. B
17	2.89	2.72	0.38	0.21	-0.284	.294	A vs. B - Trend A
18	2.03	1.92	0.54	0.38	0.256	.124	A vs. B + Trend B
19	3.07	3.39	0.05	0.36	**0.672**	.029	A vs. B - Trend A
20	4.27	3.48	n.a.	0.28	**-0.333**	.070	A vs. B + Trend B
21	2.51	2.48	0.36	0.28	0.111	.618	A vs. B + Trend B
22	2.29	3.22	0.19	0.52	**0.847**	.001	A vs. B - Trend A
23	4.00	4.24	0.13	0.45	**0.508**	.001	A vs. B + Trend B
24	3.58	3.96	0.31	0.21	**0.589**	.000	A vs. B - Trend A + Trend B

25	3.73	3.89	0.17	0.22	**0.481**	.027	A vs. B + Trend B
26	3.21	3.69	0.05	0.41	**0.667**	.083	A vs. B
27	3.61	4.16	0.41	0.22	**0.444**	.007	A vs. B - Trend A + Trend B
28	2.30	3.91	n.a.	0.49	**0.808**	.000	A vs. B + Trend B
29	2.50	1.59	n.a.	0.44	**-0.578**	.020	A vs. B + Trend B
30	3.40	3.69	0.44	0.36	0.284	.350	A vs. B - Trend A
31	3.90	3.49	0.22	0.57	**-0.559**	.001	A vs. B + Trend B
32	3.15	3.64	0.53	0.39	**0.552**	.073	A vs. B - Trend A
33	3.30	4.24	n.a.	0.54	**0.604**	.003	A vs. B + Trend B
34	3.00	3.36	0.13	0.18	**0.462**	.038	A vs. B + Trend B
35	3.00	2.91	n.a.	0.29	-0.309	.186	A vs. B + Trend B
36	2.85	3.04	0.46	0.43	0.293	.242	A vs. B - Trend A
37	3.89	4.00	0.14	0.21	0.425	.229	A vs. B
38	3.39	3.57	0.30	0.19	0.375	.185	A vs. B
39	3.79	3.58	0.57	0.37	-0.400	.117	A vs. B - Trend A
40	2.79	2.60	0.11	0.18	**-0.591**	.051	A vs. B - Trend A
41	2.80	3.09	0.03	0.17	**0.467**	.008	A vs. B + Trend B
42	2.98	3.00	0.75	0.63	0.129	.677	A vs. B

Note. Effect sizes significant on $\alpha < 0.10$ level were printed in bold type; n.a. = not available.

Table S5

*Regression Model for Tau-*U *Body Awareness Estimates as Dependent Variable and Condition, Total Practice Time, Age, Gender, Occupation, and Baseline Length as Predictors (*df *= 33)*

Variable	*b*	β	*SE*	*t*	*p*
(Intercept)	0.16	0.00	0.45	0.36	.719
ME condition	0.04	0.04	0.23	0.19	.426
MY condition	0.12	0.11	0.22	0.54	.298
MYE condition	-0.15	-0.15	0.28	-0.54	.296
Total practice time	0.00	0.23	0.00	1.03	.313
Age	-0.01	-0.19	0.01	-0.81	.422
Gender	-0.04	-0.04	0.20	-0.22	.826
Occupation	0.38	0.30	0.30	1.25	.221
Baseline length	0.01	0.11	0.01	0.63	.535

Table S6

*Regression Model for Tau-*U *Body Awareness Estimates as Dependent Variable and Component, Total Practice Time, Age, Gender, Occupation, and Baseline Length as Predictors (*df *= 34)*

Variable	*b*	β	*SE*	*t*	*p*
(Intercept)	0.32	0.00	0.43	0.74	.463
Ethical education (yes/no)	-0.11	-0.12	0.18	-0.60	.278
Physical yoga (yes/no)	-0.02	-0.02	0.18	-0.10	.460
Total practice time	0.00	0.19	0.00	0.87	.391
Age	-0.01	-0.24	0.01	-1.03	.311
Gender	-0.03	-0.02	0.20	-0.13	.894
Occupation	0.45	0.36	0.29	1.55	.131
Baseline length	0.01	0.10	0.01	0.61	.543

Table S7

Multilevel Regression Estimates for Body Awareness Scores as Dependent Variable and Time, Condition, Total Practice Time, Age, Gender, Occupation, and Baseline Length as Predictors

Variable	β	*SE*	*df*	*t*	*p*
Time	0.15	0.05	677	3.21	.001
ME condition	-0.11	0.21	33	-0.52	.605
MY condition	0.02	0.19	33	0.09	.927
MYE condition	-0.03	0.24	33	-0.14	.891
Total practice time	-0.08	0.19	33	-0.45	.655
Age	0.14	0.19	33	0.76	.451
Gender	-0.01	0.14	33	-0.08	.936
Occupation	-0.15	0.20	33	-0.74	.462
Baseline Length	-0.12	0.14	33	-0.81	.426
Time * ME condition	0.04	0.06	677	0.71	.240
Time * MY condition	0.07	0.06	677	1.13	.128
Time * MYE condition	0.02	0.06	677	0.36	.358

Table S8

Multilevel Regression Estimates for Body Awareness Scores as Dependent Variable and Time, Component, Total Practice Time, Age, Gender, Occupation, and Baseline Length as Predictors

Variable	β	*SE*	*df*	*t*	*p*
Time	0.15	0.05	678	3.17	.001
Ethical education (yes/no)	-0.10	0.18	34	-0.54	.590
Physical yoga (yes/no)	0.04	0.18	34	0.21	.833
Total practice time	-0.06	0.18	34	-0.34	.734
Age	0.16	0.18	34	0.87	.388
Gender	-0.02	0.14	34	-0.15	.883
Occupation	-0.17	0.19	34	-0.90	.376
Baseline Length	-0.12	0.14	34	-0.81	.422
Time * Ethical education	0.00	0.05	678	-0.06	.476
Time * Physical yoga	0.02	0.05	678	0.53	.299

Table S9

*Mean Difficulties with Emotion Regulation Scores With Standard Deviations for Baseline (A) and Treatment (B) Phases, and Tau-*U *Estimates With Respective Significance Levels and Type of Tau-*U *for Each Participant*

Case	*Mean* A	*Mean* B	*SD* A	*SD* B	Tau-*U*	Tau-*U* Significance	Tau-*U* Type
1	2.54	1.89	0.29	0.43	**-0.647**	.152	A vs. B - Trend A
2	2.33	2.00	0.12	0.24	**-0.500**	.042	A vs. B + Trend B
3	3.75	3.33	n.a.	0.20	-0.286	.362	A vs. B + Trend B
4	1.50	1.85	n.a.	0.31	0.414	.251	A vs. B + Trend B
5	1.38	1.31	0.18	0.14	-0.231	.603	A vs. B - Trend A
6	3.42	3.02	0.14	0.23	**-0.852**	.033	A vs. B
7	missing						
8	3.42	3.03	n.a.	0.19	-0.476	.129	A vs. B + Trend B
9	2.79	2.07	0.53	0.38	**-0.800**	.001	A vs. B - Trend A + Trend B
10	1.71	1.75	0.06	0.25	0.023	.927	A vs. B + Trend B
11	2.00	1.66	n.a.	0.10	0.056	.831	A vs. B + Trend B
12	2.39	1.98	0.35	0.42	**-0.709**	.002	A vs. B - Trend A + Trend B
13	2.08	1.93	0.12	0.20	**-0.523**	.037	A vs. B + Trend B
14	2.50	1.81	n.a.	0.57	**-0.905**	.003	A vs. B + Trend B
15	1.08	1.17	n.a.	0.11	-0.393	.111	A vs. B + Trend B
16	1.75	1.81	0.33	0.18	0.200	.580	A vs. B - Trend A
17	3.06	2.84	0.34	0.25	**-0.524**	.022	A vs. B + Trend B
18	1.81	1.87	0.46	0.37	-0.111	.649	A vs. B - Trend A + Trend B
19	2.71	2.21	0.18	0.51	**-0.765**	.088	A vs. B - Trend A
20	2.25	2.05	n.a.	0.29	**-0.500**	.083	A vs. B + Trend B
21	2.96	2.67	0.29	0.20	**-0.667**	.163	A vs. B - Trend A
22	3.61	2.91	0.17	0.25	**-0.697**	.001	A vs. B - Trend A + Trend B
23	2.67	1.81	n.a.	0.46	**-0.822**	.001	A vs. B + Trend B
24	2.25	2.14	0.46	0.27	**-0.379**	.083	A vs. B - Trend A + Trend B

25	3.00	2.47	0.24	0.18	**-0.571**	.044	A vs. B - Trend A + Trend B
26	2.54	2.13	0.18	0.33	**-0.714**	.024	A vs. B - Trend A + Trend B
27	2.33	2.27	0.35	0.38	**-0.422**	.083	A vs. B - Trend A + Trend B
28	missing						
29	missing						
30	2.67	2.69	0.59	0.33	0.200	.382	A vs. B - Trend A + Trend B
31	1.78	1.65	0.38	0.37	-0.327	.160	A vs. B - Trend A + Trend B
32	3.46	3.11	0.29	0.46	**-0.500**	.083	A vs. B - Trend A + Trend B
33	3.92	2.86	n.a.	0.62	**-0.750**	.009	A vs. B + Trend B
34	2.58	2.67	n.a.	0.20	-0.200	.602	A vs. B + Trend B
35	2.33	2.27	n.a.	0.17	**-0.700**	.077	A vs. B + Trend B
36	2.22	1.83	0.32	0.34	-0.433	.234	A vs. B - Trend A
37	2.83	2.30	0.12	0.41	**-0.611**	.020	A vs. B - Trend A + Trend B
38	1.86	2.14	0.21	0.28	0.567	.118	A vs. B - Trend A
39	2.11	2.30	0.27	0.67	0.080	.798	A vs. B + Trend B
40	1.42	1.44	0.00	0.18	-0.318	.193	A vs. B + Trend B
41	3.42	2.73	n.a.	0.33	**-0.528**	.046	A vs. B + Trend B
42	2.44	2.30	0.29	0.19	-0.333	.171	A vs. B - Trend A + Trend B

Note. Effect sizes significant on $\alpha < 0.10$ level were printed in bold type; n.a. = not available.

Table S10

Regression Model for Tau-U *Difficulties with Emotion Regulation Estimates as Dependent Variable and Condition, Total Practice Time, Age, Gender, Occupation, and Baseline Length as Predictors (*df *= 30)*

Variable	*b*	β	*SE*	*t*	*p*
(Intercept)	-0.98	0.00	0.40	-2.47	.019
ME condition	-0.03	-0.03	0.19	-0.13	.448
MY condition	-0.16	-0.19	0.20	-0.81	.211
MYE condition	-0.01	-0.01	0.24	-0.05	.482
Total practice time	0.00	0.09	0.00	0.38	.706
Age	0.01	0.33	0.01	1.27	.212
Gender	-0.06	-0.07	0.16	-0.37	.714
Occupation	-0.17	-0.18	0.26	-0.67	.511
Baseline length	0.01	0.22	0.01	1.21	.237

Table S11

Regression Model for Tau-U *Difficulties with Emotion Regulation Estimates as Dependent Variable and Component, Total Practice Time, Age, Gender, Occupation, and Baseline Length as Predictors (*df *= 31)*

Variable	*b*	β	*SE*	*t*	*p*
(Intercept)	-1.04	0.00	0.38	-2.72	.011
Ethical education (yes/no)	0.06	0.08	0.15	0.38	.353
Physical yoga (yes/no)	-0.08	-0.11	0.16	-0.48	.316
Total practice time	0.00	0.11	0.00	0.46	.649
Age	0.01	0.36	0.01	1.38	.178
Gender	-0.07	-0.08	0.16	-0.43	.670
Occupation	-0.21	-0.21	0.25	-0.83	.415
Baseline length	0.01	0.21	0.01	1.17	.250

Table S12

Multilevel Regression Estimates for Difficulties with Emotion Regulation Scores as Dependent Variable and Time, Condition, Total Practice Time, Age, Gender, Occupation, and Baseline Length as Predictors

Variable	β	*SE*	*df*	*t*	*p*
Time	-0.26	0.05	334	-5.55	.000
ME condition	-0.03	0.19	33	-0.16	.870
MY condition	0.15	0.18	33	0.84	.408
MYE condition	0.33	0.22	33	1.48	.148
Total practice time	-0.33	0.17	33	-1.91	.066
Age	0.00	0.17	33	0.01	.991
Gender	-0.21	0.13	33	-1.58	.123
Occupation	-0.06	0.18	33	-0.30	.762
Baseline Length	0.07	0.13	33	0.55	.586
Time * ME condition	-0.01	0.06	334	-0.17	.431
Time * MY condition	-0.07	0.06	334	-1.20	.116
Time * MYE condition	-0.01	0.06	334	-0.23	.408

Table S13

Multilevel Regression Estimates for Difficulties with Emotion Regulation Scores as Dependent Variable and Time, Component, Total Practice Time, Age, Gender, Occupation, and Baseline Length as Predictors

Variable	β	*SE*	*df*	*t*	*p*
Time	-0.26	0.05	335	-5.59	.000
Ethical education (yes/no)	0.08	0.16	34	0.48	.632
Physical yoga (yes/no)	0.27	0.16	34	1.66	.107
Total practice time	-0.30	0.17	34	-1.73	.092
Age	0.03	0.17	34	0.20	.843
Gender	-0.23	0.13	34	-1.71	.096
Occupation	-0.10	0.18	34	-0.59	.560
Baseline Length	0.07	0.13	34	0.52	.606
Time * Ethical education	0.03	0.05	335	0.59	.278
Time * Physical yoga	-0.04	0.05	335	-0.86	.196

Table S14

Mean Valence and Arousal Scores With Standard Deviations for Baseline (A) and Treatment (B) Phases, and Tau-U Estimates and Categorized Affective Response for Each Participant

Case	Valence			Arousal			Response
	Mean A	Mean B	Tau-U	Mean A	Mean B	Tau-U	
1	4.83	5.38	0.175	5.58	6.23	0.229	more energetic
2	3.93	4.00	**-0.197**	6.21	6.00	-0.093	more stressed
3	4.29	4.09	-0.091	6.12	6.41	0.123	more stressed
4	5.14	3.91	**-0.392**	8.43	8.16	-0.241	more stressed
5	4.70	4.29	-0.250	6.70	6.76	0.035	more stressed
6	4.14	4.17	**0.175**	7.00	6.64	-0.117	more energetic
7	3.60	3.34	**-0.206**	6.60	6.60	0.045	more stressed
8	2.67	3.43	0.337	6.17	6.46	0.097	less stressed
9	4.35	3.96	-0.134	5.41	6.41	**0.397**	more stressed
10	5.07	4.80	-0.142	6.21	6.48	0.097	no change
11	4.00	3.95	-0.011	5.71	6.49	**0.474**	more stressed
12	4.06	4.70	0.245	5.94	6.30	**0.229**	more energetic
13	3.62	4.37	**0.373**	5.92	6.00	0.054	more energetic
14	4.67	4.63	0.129	6.50	7.02	**0.184**	more energetic
15	6.27	6.84	**0.360**	4.91	4.34	**-0.332**	more relaxed
16	4.24	4.44	0.082	6.52	6.04	**-0.253**	more relaxed
17	4.20	3.93	-0.188	6.55	6.04	-0.213	less stressed
18	4.43	5.50	**0.334**	6.48	5.34	**-0.268**	more relaxed
19	4.69	4.02	**-0.220**	5.92	6.30	0.133	more stressed
20	4.20	4.73	0.103	6.00	6.49	0.111	more energetic
21	3.92	4.00	-0.156	6.46	6.83	-0.137	more stressed
22	4.00	3.96	-0.046	6.53	6.71	0.006	no change
23	4.71	4.46	-0.138	7.00	7.03	0.002	no change
24	4.47	4.28	-0.115	6.88	6.70	-0.073	no change
25	3.83	3.81	-0.076	7.06	6.78	-0.120	less stressed
26	3.85	4.35	0.260	7.23	6.53	**-0.346**	more energetic
27	4.50	4.61	0.038	7.25	6.39	**-0.262**	more relaxed
28	5.00	4.13	-0.211	6.00	6.06	0.000	more energetic

29	3.40	3.89	0.068	5.80	6.11	0.177	no change
30	4.54	5.11	0.186	6.23	6.59	0.177	more energetic
31	4.38	4.19	0.071	6.81	7.00	-0.032	no change
32	3.86	3.85	**-0.189**	6.14	6.92	**0.357**	more stressed
33	4.40	4.86	0.115	7.00	6.82	-0.121	more relaxed
34	3.55	4.44	0.256	6.82	6.56	-0.119	more energetic
35	3.83	4.24	0.162	7.00	6.50	-0.272	more energetic
36	4.55	4.89	-0.025	6.50	6.84	0.152	more energetic
37	4.45	4.54	0.040	6.09	6.64	0.209	more energetic
38	5.00	4.12	**-0.253**	6.90	7.82	**0.379**	more stressed
39	4.16	3.67	-0.205	7.42	6.59	**-0.300**	more stressed
40	5.64	5.52	-0.091	6.79	6.22	-0.247	more relaxed
41	4.14	4.13	0.027	5.71	6.38	0.268	more energetic
42	3.76	4.64	0.178	6.35	6.06	-0.092	more relaxed

Note. Effect sizes significant on α < 0.10 level were printed in bold type.

Table S15

*Regression Model for Tau-*U *Valence Estimates as Dependent Variable and Condition, Total Practice Time, Age, Gender, Occupation, and Baseline Length as Predictors (*df *= 33)*

Variable	*b*	β	*SE*	*t*	*p*
(Intercept)	-0.06	0.00	0.20	-0.28	.780
ME condition	0.18	0.41	0.10	1.80	.041
MY condition	0.08	0.18	0.10	0.82	.208
MYE condition	0.09	0.20	0.12	0.70	.245
Total practice time	0.00	-0.07	0.00	-0.32	.753
Age	0.00	0.05	0.01	0.21	.838
Gender	0.04	0.07	0.09	0.43	.670
Occupation	-0.07	-0.13	0.14	-0.54	.594
Baseline length	0.00	-0.04	0.01	-0.24	.810

Table S16

*Regression Model for Tau-*U *Valence Estimates as Dependent Variable and Component, Total Practice Time, Age, Gender, Occupation, and Baseline Length as Predictors (*df *= 34)*

Variable	*b*	β	*SE*	*t*	*p*
(Intercept)	0.03	0.00	0.19	0.16	.875
Ethical education (yes/no)	0.10	0.25	0.08	1.22	.230
Physical yoga (yes/no)	0.00	0.01	0.08	0.05	.964
Total practice time	0.00	-0.12	0.00	-0.53	.601
Age	0.00	-0.01	0.01	-0.05	.962
Gender	0.05	0.09	0.09	0.54	.595
Occupation	-0.03	-0.05	0.13	-0.22	.828
Baseline length	0.00	-0.04	0.01	-0.26	.800

Table S17

Multilevel Regression Estimates for Valence Scores as Dependent Variable and Time, Condition, Total Practice Time, Age, Gender, Occupation, and Baseline Length as Predictors

Variable	β	*SE*	*df*	*t*	*p*
Time	0.00	0.03	2492.00	-0.08	.469
ME condition	0.06	0.09	33.00	0.63	.532
MY condition	-0.03	0.08	33.00	-0.31	.759
MYE condition	-0.08	0.10	33.00	-0.79	.432
Total practice time	0.17	0.08	33.00	2.23	.033
Age	-0.02	0.08	33.00	-0.29	.774
Gender	0.11	0.06	33.00	1.74	.092
Occupation	-0.05	0.08	33.00	-0.61	.544
Baseline Length	0.00	0.06	33.00	0.08	.938
Time * ME condition	0.08	0.03	2492.00	2.23	.013
Time * MY condition	0.06	0.03	2492.00	1.84	.033
Time * MYE condition	0.04	0.03	2492.00	1.23	.110

Table S18

Multilevel Regression Estimates for Valence Scores as Dependent Variable and Time, Component, Total Practice Time, Age, Gender, Occupation, and Baseline Length as Predictors

Variable	β	*SE*	*df*	*t*	*p*
Time	0.00	0.03	2493.00	-0.13	.448
Ethical education (yes/no)	0.00	0.08	34.00	-0.01	.995
Physical yoga (yes/no)	-0.10	0.08	34.00	-1.25	.221
Total practice time	0.17	0.08	34.00	2.26	.031
Age	-0.03	0.08	34.00	-0.34	.736
Gender	0.10	0.06	34.00	1.76	.088
Occupation	-0.05	0.08	34.00	-0.58	.566
Baseline Length	0.00	0.06	34.00	0.07	.945
Time * Ethical education	0.03	0.03	2493.00	1.08	.140
Time * Physical yoga	0.01	0.03	2493.00	0.52	.300

Table S19

*Regression Model for Tau-*U *Arousal Estimates as Dependent Variable and Condition, Total Practice Time, Age, Gender, Occupation, and Baseline Length as Predictors (*df *= 32)*

Variable	*b*	β	*SE*	*t*	*p*
(Intercept)	0.41	0.00	0.21	1.98	.056
ME condition	-0.13	-0.27	0.10	-1.28	.104
MY condition	-0.12	-0.25	0.10	-1.24	.112
MYE condition	-0.05	-0.10	0.12	-0.39	.350
Total practice time	0.00	0.03	0.00	0.14	.893
Age	-0.01	-0.31	0.01	-1.56	.129
Gender	-0.13	-0.23	0.09	-1.48	.149
Occupation	0.29	0.45	0.14	2.16	.038
Baseline length	-0.01	-0.16	0.01	-1.02	.313

Table S20

*Regression Model for Tau-*U *Arousal Estimates as Dependent Variable and Component, Total Practice Time, Age, Gender, Occupation, and Baseline Length as Predictors (*df *= 33)*

Variable	*b*	β	*SE*	*t*	*p*
(Intercept)	0.32	0.00	0.20	1.58	.123
Ethical education (yes/no)	-0.03	-0.08	0.08	-0.41	.344
Physical yoga (yes/no)	-0.03	-0.07	0.08	-0.39	.350
Total practice time	0.00	0.08	0.00	0.41	.688
Age	-0.01	-0.29	0.01	-1.40	.171
Gender	-0.14	-0.25	0.09	-1.55	.131
Occupation	0.24	0.37	0.13	1.82	.079
Baseline length	-0.01	-0.15	0.01	-0.96	.345

Table S21

Multilevel Regression Estimates for Arousal Scores as Dependent Variable and Time, Condition, Total Practice Time, Age, Gender, Occupation, and Baseline Length as Predictors

Variable	β	*SE*	*df*	*t*	*p*
Time	0.00	0.03	2454	0.15	.883
ME condition	-0.06	0.06	32	-0.94	.353
MY condition	0.05	0.06	32	0.89	.381
MYE condition	0.07	0.08	32	0.92	.363
Total practice time	0.01	0.06	32	0.24	.814
Age	0.08	0.06	32	1.18	.247
Gender	-0.08	0.04	32	-1.75	.090
Occupation	-0.06	0.06	32	-0.97	.338
Baseline Length	0.05	0.05	32	1.01	.321
Time * ME condition	-0.03	0.04	2454	-0.74	.228
Time * MY condition	-0.02	0.04	2454	-0.48	.315
Time * MYE condition	0.01	0.04	2454	0.28	.390

Table S22

Multilevel Regression Estimates for Arousal Scores as Dependent Variable and Time, Component, Total Practice Time, Age, Gender, Occupation, and Baseline Length as Predictors

Variable	β	*SE*	*df*	*t*	*p*
Time	0.00	0.03	2455	0.15	.440
Ethical education (yes/no)	-0.03	0.06	33	-0.47	.639
Physical yoga (yes/no)	0.10	0.06	33	1.88	.069
Total practice time	0.02	0.06	33	0.34	.738
Age	0.08	0.06	33	1.27	.212
Gender	-0.08	0.04	33	-1.77	.085
Occupation	-0.07	0.06	33	-1.18	.248
Baseline Length	0.05	0.04	33	1.06	.297
Time * Ethical education	0.00	0.03	2455	0.05	.479
Time * Physical yoga	0.01	0.03	2455	0.45	.327

Table S23

Frequencies of Categorized Affective Reponses to the Treatment in Each Condition

Affective Response	MA	ME	MY	MYE	Sum
More Energetic	2	4	2	5	14
More Relaxed	0	3	1	3	7
Less Stressed	1	1	1	0	3
No Change	1	0	6	0	6
More Stressed	6	3	0	3	12
